# Internet-based cognitive behavioural therapy as a feasible treatment of adult-onset, focal, isolated, idiopathic cervical dystonia

**DOI:** 10.1016/j.prdoa.2021.100121

**Published:** 2021-11-27

**Authors:** Megan E. Wadon, Claire MacIver, Mia Winter, Kathryn J. Peall

**Affiliations:** a Neuroscience and Mental Health Research Institute, Hadyn Ellis Building, Cardiff University, Cardiff, UK; b Cardiff University Brain Research Imaging Centre, Cardiff University, Cardiff, UK; c Department of Clinical Neuropsychology, University Hospital of Wales, Cardiff, UK

**Keywords:** Feasibility studies, Cognitive behavioural therapy, Anxiety, Depression, Dystonia

## Abstract

**Introduction:**

Psychiatric symptoms are well recognised co-morbid traits in adult-onset idiopathic, isolated, focal cervical dystonia (AOIFCD), although few studies have sought to address their management. Internet-based cognitive behavioural therapy (iCBT) may provide an accessible solution. Here, we determine the feasibility of using iCBT in the management of non-motor symptoms for individuals with AOIFCD.

**Methods:**

Participants were randomised to receive an 8-week iCBT programme (n = 10) or not (n = 10), both alongside routine clinical care. All participants underwent assessments at baseline, 3-, and 6- months for anxiety, depression, quality of life and motor symptoms, and engagement with iCBT was recorded. Group differences over time were determined using two-way mixed ANOVA, and simple statistics evaluated change on an individual participant level.

**Results:**

Over half of participants receiving iCBT (6/10) showed high engagement, with feedback indicating most participants found iCBT useful (6/8), would continue to use it (7/8), and try it again if offered (7/8). Although no between-group significant differences were observed (e.g. Beck’s Depression Inventory p = 0.067) anxiety and depression levels showed trends towards improvement at 3-months in those receiving iCBT. Individual level analysis also indicated higher percentage level improvements in these symptoms, with this sustained in 86% participants.

**Conclusion:**

iCBT represents a feasible therapeutic option in the management of co-morbid anxiety and depression in AOIFCD. Further work is needed to replicate these findings in a larger cohort, identify those most likely to benefit from this form of therapy and overcome barriers hindering those less likely to engage with this form of treatment.

## Highlights

• Internet-based CBT is feasible for individuals with adult-onset cervical dystonia.
• Internet-based CBT reduces depression and anxiety in adult-onset cervical dystonia.
• Effects from internet-based CBT are sustained in some individuals at six months.

## 1 Introduction

Psychiatric symptoms, in particular depression and anxiety, are increasingly recognised as part of the phenotypic spectrum of adult-onset, focal, isolated, idiopathic cervical dystonia (AOIFCD) [1]. In spite of this, there remains no standardised management strategy, with available pharmacological treatment often exacerbating the underlying movement disorder [2]. These psychiatric symptoms have also been shown to have a greater impact on quality of life (QoL) than the motor symptoms themselves, further reinforcing the need to develop appropriate treatment options [1].

Previous case reports have demonstrated promise for cognitive behavioural therapy (CBT) in managing anxiety and depression in AOIFCD [3], [4], however timely access of face-to-face psychological therapy is often limited by cost, waiting times and a shortage of suitably qualified therapists. This has resulted in a number of internet-based CBT (iCBT) programmes being developed, with many focused on the management of depression and anxiety [5], [6].

This study demonstrates the feasibility of using an anxiety and depression focused iCBT programme for individuals with AOIFCD, as well as determining its impact on these symptoms, motor symptom severity and QoL. iCBT has the potential to provide an accessible, cost-effective care model that could be offered alongside currently available medical management, maximising the use of available healthcare resources.

## 2 Methods

**Fig. 1:**
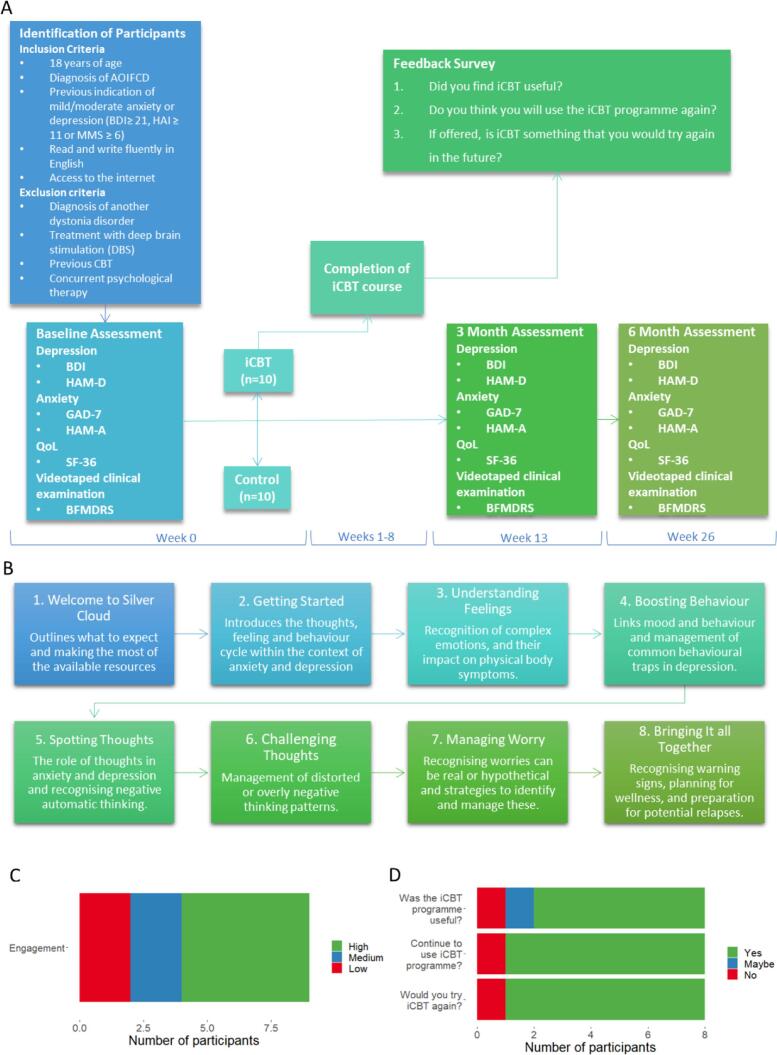
A flow chart detailing recruitment of participants and participant activities, along with the time-points they occurred in the study (A). Descriptions of the iCBT modules participants were asked to complete, in the sequence in which they were advised to complete them (B). Engagement levels of participants receiving iCBT (C), and the responses to the feedback questions about the iCBT programme (D). AOIFCD, adult-onset, focal, isolated, idiopathic cervical dystonia; BDI, Beck’s Depression Inventory; BFMDRS, Burke-Fahn-Marsden Dystonia Rating Scale, GAD-7, Generalised Anxiety Disorder-7; HAM-A, Hamilton Scale for Anxiety; HAM-D, Hamilton Scale for Depression; iCBT, internet-based cognitive behavioural therapy; SF-36, Short Form-36 Health Survey.

Participants were recruited, providing informed consent in paper format or via an online platform (Research Ethics committee reference: 19/WA/0265), following a previously detailed protocol (Fig. 1a) [7]. Randomisation was completed on a 1:1 ratio using sealed opaque envelopes that contained a computer-generated random allocation code. Envelope selection was made by a blind assessor and were opened during the participants baseline assessment. Due to the nature of the intervention, it was not possible to blind the participant to the outcome of the randomisation. Participants randomised into the iCBT intervention group were introduced to the iCBT platform, hosted by SilverCloud Health Ltd (www.silvercloudhealth.com), provided with a link to the “Space from Anxiety and Depression” programme, and asked to complete the course (one module a week) over the subsequent 8 weeks (Fig. 1b).

Our primary outcome measure was the extent of participant engagement with the programme, with the frequency of ‘logins’ recorded, and participants classified as having high (minimum of 7 weeks of programme activity), medium (active for 4–7 weeks), or low (active for < 4 weeks) engagement over the 8-week period. Those recruited to the iCBT arm were also asked to respond to a short feedback survey (Fig. 1a).

Secondary outcome measures assessed for changes in motor and non-motor symptomatology at baseline, 3-months, and 6-months post enrolment. These were conducted in the participant’s home, university research clinic or online using videoconferencing software (Fig. 1a). Motor symptoms were scored using the Burke-Fahn-Marsden Dystonia Rating Scale (BFMDRS) by two independent movement disorder specialists blind to the participants group allocation, with an average of these scores combined with the participant completed BFMDRS Disability Scale. Detailed psychiatric evaluation was also conducted at each timepoint via the MINI International Neuropsychiatric Interview, with anonymised individual participant level data available in the supplementary material (Supplementary Tables 1, 2 and 3).

Statistical analysis was conducted using R version 3.6.3 [8]. Data on participant responses to iCBT is reported as frequencies. Two-way mixed ANOVA determined differences between the iCBT and control groups over time. Participants were excluded from onward analysis if they demonstrated low programme engagement or if not all 3 assessments were complete. Percentage change from baseline for each participant was also reported for each symptom outcome.

## 3 Results

Twenty participants (15 female, 5 male), aged 42–72 years (median: 57.5 years) were recruited. Ten participants received the iCBT intervention alongside routine clinical care (6 female, 4 male, median age 61 years (46–72 years)), while 10 continued to receive their ongoing clinical care (9 female, 1 male, median age 57 years (42–67 years)). Sixteen participants completed all assessments at 3-months (8 iCBT, 8 control) and 6-months (7 iCBT, 9 control). Two participants were taking medications classified as antidepressants or anxiolytics at over the duration of the study, one of which was randomised to receive iCBT, and one randomised to the control group. Over half of those receiving iCBT (6/10, 60%) showed high engagement with the programme, with two (20%) each demonstrating medium and low engagement (Fig. 1c). Eight completed the iCBT feedback questionnaire, with 6/8 (75%) describing the programme as useful, 1/8 (12.5%) unsure, and 1/8 (12.5%) not finding the programme useful. Seven of the eight participants (87.5%) indicated they would continue to use the iCBT programme, and seven (87.5%) indicated they would try iCBT again if offered (Fig. 1d).

**Fig. 2:**
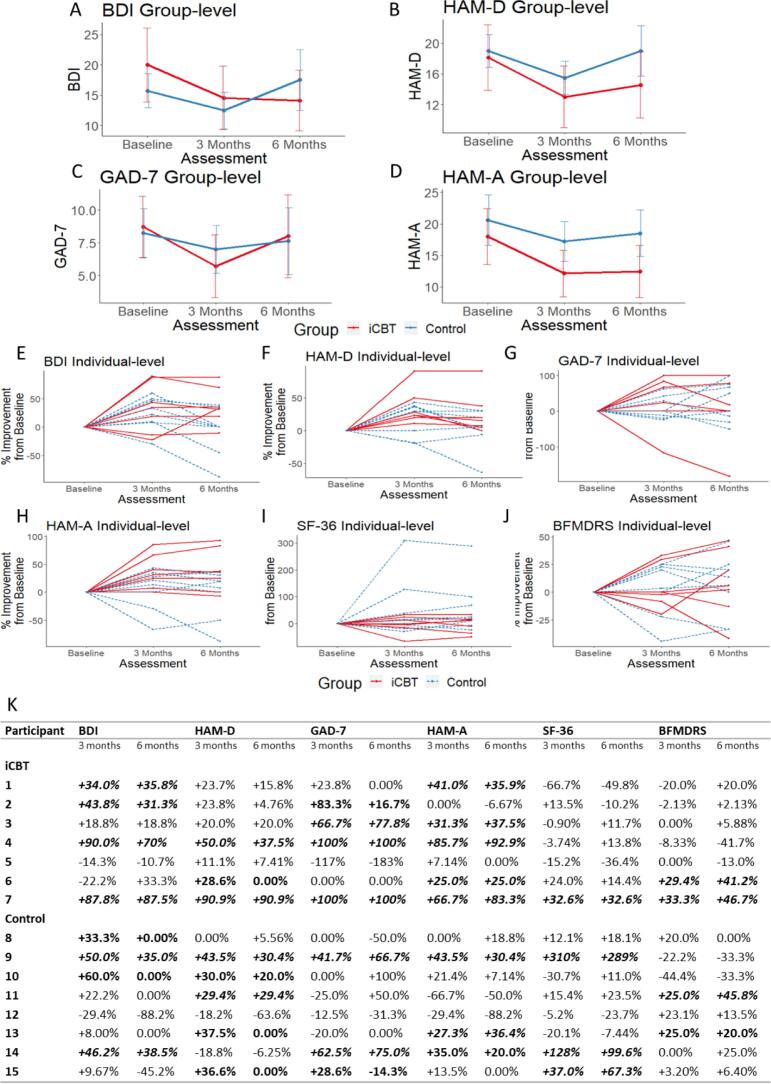
Symptom effects in the iCBT and control groups for group-level BDI effects (A), group-level HAM-D effects (B), group-level GAD-7 effects (C), group-level HAM-A effects (D), individual level BDI effects (E), individual level HAM-D effects (F), individual level GAD-7 effects (G), individual level HAM-A effects (H), individual level SF-36 effects (I), individual level BFMDRS effects (J), and individual level improvement from baseline for anxiety, depression, QoL, and motor scores (K). Positive (+) changes indicate improvement in outcome scores. **Bold** indicates ≥ 25% improvement from baseline at 3-months, whilst ***bold italics*** indicates a sustained improvement of ≥ 25% from baseline at 6-months as well as 3-months. Group-level graphs (A-D) show the mean raw score for each questionnaire, with error bars representing the standard error, and lower scores indicating an improvement in symptoms. Individuals-level graphs (E-J) represent the percentage change from baseline for each individual participant, with positive changes indicating an improvement in symptoms. BDI, Beck’s Depression Inventory; BFMDRS, Burke-Fahn-Marsden Dystonia Rating Scale, GAD-7, Generalised Anxiety Disorder-7; HAM-A, Hamilton Scale for Anxiety; HAM-D, Hamilton Scale for Depression; iCBT, internet-based cognitive behavioural therapy; SF-36, Short Form-36 Health Survey.

Based on programme engagement and assessment completion, 7/10 iCBT and 8/10 control participants were included for onward analysis. No significant difference in depression or anxiety-related symptoms were observed between the two groups (Supplementary Table 4). There was a greater trend towards depression and anxiety score improvement in those receiving iCBT at 3-months, with this improvement sustained at 6-months for measures of depression (Fig. 2a & b). However, anxiety-related measures demonstrated mixed results with Hamilton Scale for Anxiety (HAM-A) scores showing a sustained effect (Fig. 2c), but Generalised Anxiety Disorder-7 (GAD-7) scores returning towards baseline (Fig. 2d). Interestingly, although there was no significant difference between groups, there was a statistically significant improvement in depression scores between baseline and 3-months (Beck Depression Inventory (BDI) p = 0.04; Hamilton Scale for Depression (HAM-D) p = 0.008), and anxiety-related symptoms measured by the HAM-A (p = 0.043). QoL measures (p = 0.416) and motor impairment (p = 0.880) demonstrated no statistically significant differences between the groups over the 6-month period.

Individual level analysis demonstrated improvements across multiple symptom groups in 6/7 of those receiving iCBT and 7/8 of those in the control group (Fig. 2e–k). At 3-months, percentage improvements across multiple domains were higher for those receiving the iCBT compared to controls; BDI (iCBT 90.0%, control 60%, Fig. 2e, HAM-D (iCBT 90.9%, control 43.5%, Fig. 2f, GAD-7 (iCBT 100%, control 62.5%, Fig. 2g, HAM-A (iCBT 85.7%, control 43.5%, Fig. 2h, and motor impairment (iCBT 33.3%, control 25.5%, Fig. 2j. Using a threshold of ≥ 25% improvement at both 3- and 6-months, again higher levels of sustained improvement were observed amongst those receiving iCBT with measures of depression (BDI: iCBT 4/7, control 2/8; HAM-D: iCBT 2/7, control 2/8), anxiety (GAD-7: iCBT 3/7, control 2/8; HAM-A: iCBT 5/7, control 2/8), and motor impairment (BFMDRS: iCBT 2/7, control 1/8).

## 4 Discussion

This study demonstrates the feasibility of iCBT in the management of anxiety and depression for those diagnosed with AOIFCD. Sixty percent of those receiving iCBT demonstrated high engagement, with 75% of feedback responses indicating its utility and 87.5% indicating they would continue to use the programme and/or try another iCBT programme. Although no statistically significant differences were observed, those receiving the iCBT intervention also demonstrated a trend towards improvement in anxiety and depression at 3 months post-enrolment, with sustained effects in some individuals at 6-months, supported by larger improvements, and more sustained, individual-level percentage improvements.

The lack of statistically significant between group differences in reported symptoms is likely due to the small sample size of this study. Trends towards improvement were observed in depression and anxiety scores, although we did not see any improvement in QoL or motor impairment. Interestingly, we saw general improvements in depression and anxiety between baseline and 3-months across both study groups. This may be due to volunteer bias, as participants may have been more likely to volunteer to take part in the study if they felt their psychiatric symptoms were worse than usual, or due to the COVID-19 pandemic which occurred during the data collection phase of this study, with 12 participants undergoing baseline assessments prior to national lockdowns being introduced, and the remaining 8 recruited following lockdown introductions. In the general population, the COVID-19 pandemic had a detrimental effect on mental health [9], although this did seem to recover [10] with some evidence suggesting individuals with certain medical conditions had reduced psychological distress [11], particularly given the high reported rate of social anxiety amongst those with AOIFCD [12]. This may also have had an impact on QoL scores, particularly relating to the uncertainty around regular receipt of neurotoxin injections, recognised as providing a positive impact on QoL for individuals with AOIFCD [13], possibly providing some explanation for the variation observed across both groups on an individual level.

Previous studies involving face-to-face CBT have shown sustained positive impact for those with AOIFCD beyond 6 months [4]. In this study, while measures of depression appeared to indicate sustained improvement, results from anxiety-focused questionnaires were more variable. The programme we used was relatively short with only eight modules and no requirement to revise any sections after course completion, therefore it may be that a longer programme, with refresher sessions built in may produce more consistent sustained improvement.

Although the overall response to iCBT was positive, some individuals did not engage, and a small proportion gave negative feedback. We also saw large variation in individual symptom effects, with some participants demonstrating very little or no symptom improvement. This suggests iCBT may not be an appropriate management strategy for all individuals with AOIFCD, with suitability dependent on additional factors not included in this study. Several factors have been identified as potential barriers to iCBT including computer anxiety, self-stigma, and lower perceived need [14].

In conclusion, iCBT provides a feasible option in the management of symptoms of anxiety and depression in those diagnosed with AOIFCD. Further investigation in larger sample sizes is needed to fully determine the symptom effects of iCBT, identify those most likely to benefit, as well as addressing potential barriers to this intervention. Once refined, iCBT could provide an accessible, cost-effective treatment option that could be administered alongside current management strategies, maximising health care resources and addressing gaps in the current care model.

## 5 Data availability statement

The data that support the findings of this study are available from the corresponding author upon reasonable request.

## CRediT authorship contribution statement

Megan E. Wadon: Data curation, Formal analysis, Methodology, Project Administration, Visualization, Writing - original draft, Writing - review & editing. Claire MacIver: Formal Analysis, Writing - review & editing. Mia Winter: Conceptualization, Funding acquisition, Methodology, Writing - review & editing. Kathryn J. Peall: Conceptualization, Data curation, Formal analysis, Funding acqusition, Methodology, Resources, Supervision, Writing - original draft, Writing - review & editing.

## Declaration of Competing Interest

The authors declare that they have no known competing financial interests or personal relationships that could have appeared to influence the work reported in this paper.
