# Supplementary material for: Internet-based cognitive behavioural therapy as a feasible treatment of adult-onset, focal, isolated, idiopathic cervical dystonia
Source: Clin Park Relat Disord. 2021 Nov 27;5:100121. doi: 10.1016/j.prdoa.2021.100121 (PMC8649077; doi:10.1016/j.prdoa.2021.100121)
Supplement: Supplementary data 1 [file mmc1.docx]

| **Supplementary Table 1**: MINI International Neuropsychiatric Interview outcomes for mood related disorders for all participants. Blacked out boxes indicate an assessment did not take place | | | | | | | | | | | | |
| --- | --- | --- | --- | --- | --- | --- | --- | --- | --- | --- | --- | --- |
| Participant | Depression | | | | Dysthymia | | | | Hypomanic Episode | | | |
|  | Past | Baseline | 3 months | 6 months | Past | Baseline | 3 months | 6 months | Past | Baseline | 3 months | 6 months |
| iCBT |  |  |  |  |  |  |  |  |  |  |  |  |
| 1 | ✓ | ✓ | ✓ | ✓ | ✓ | ✓ | ✓ | ✓ | ✓ |  |  |  |
| 2 | ✓ | ✓ |  |  |  |  | ✓ |  |  |  |  |  |
| 3 |  |  |  |  |  |  |  |  |  |  |  |  |
| 4 | ✓ |  |  |  | ✓ |  |  |  |  |  |  |  |
| 5 | ✓ |  |  |  |  |  |  |  |  |  |  |  |
| 6 |  |  |  |  |  |  |  |  |  |  |  |  |
| 7 |  |  |  |  |  |  |  |  | ✓ |  |  |  |
| 16 | ✓ | ✓ |  |  | ✓ |  |  |  |  | ✓ |  |  |
| 17 | ✓ |  |  |  |  |  |  |  |  |  |  |  |
| 18 |  |  |  |  |  |  |  |  |  |  |  |  |
| Control |  |  |  |  |  |  |  |  |  |  |  |  |
| 8 | ✓ |  |  |  | ✓ |  |  |  |  |  |  |  |
| 9 | ✓ | ✓ |  |  |  |  | ✓ |  |  |  |  |  |
| 10 |  |  |  |  |  |  |  |  |  |  |  |  |
| 11 | ✓ |  |  |  |  |  |  |  |  |  |  |  |
| 12 | ✓ |  |  | ✓ | ✓ |  |  |  |  |  |  |  |
| 13 |  |  |  |  |  |  |  |  |  |  |  |  |
| 14 |  |  |  |  |  |  |  |  |  |  |  |  |
| 15 | ✓ | ✓ | ✓ | ✓ | ✓ |  |  | ✓ |  |  |  |  |
| 19 |  |  |  |  |  |  |  |  |  |  |  |  |
| 20 |  |  |  |  |  |  |  |  |  |  |  |  |

| **Supplementary Table 2**: MINI International Neuropsychiatric Interview outcomes for anxiety related disorders for all participants. Blacked out boxes indicate an assessment did not take place | | | | | | | | | | | | | | | | | |
| --- | --- | --- | --- | --- | --- | --- | --- | --- | --- | --- | --- | --- | --- | --- | --- | --- | --- |
| Participant | Panic Disorder | | | | Agoraphobia | | | | Social Phobia | | | Specific Phobia | | | GAD | | |
|  | Past | Baseline | 3 months | 6 months | Past | Baseline | 3 months | 6 months | Baseline | 3 months | 6 months | Baseline | 3 months | 6 months | Baseline | 3 months | 6 months |
| iCBT |  |  |  |  |  |  |  |  |  |  |  |  |  |  |  |  |  |
| 1 | ✓ |  |  |  | ✓ | ✓ | ✓ | ✓ | ✓ | ✓ | ✓ |  |  |  |  |  |  |
| 2 |  |  |  |  | ✓ |  | ✓ |  | ✓ | ✓ | ✓ |  |  |  |  |  |  |
| 3 |  |  |  |  |  |  |  |  |  |  |  |  |  |  |  |  |  |
| 4 | ✓ |  |  |  |  |  |  |  | ✓ |  |  |  |  |  |  |  |  |
| 5 |  |  |  |  | ✓ | ✓ | ✓ | ✓ | ✓ | ✓ | ✓ |  |  | ✓ | ✓ |  |  |
| 6 | ✓ |  |  |  | ✓ |  |  | ✓ |  | ✓ | ✓ |  |  |  |  |  |  |
| 7 |  |  |  |  |  |  |  |  |  |  |  |  |  |  |  |  |  |
| 16 | ✓ | ✓ |  |  |  |  |  |  | ✓ |  |  |  |  |  | ✓ |  |  |
| 17 |  |  |  |  |  |  |  |  |  |  |  |  |  |  |  |  |  |
| 18 | ✓ |  |  |  | ✓ | ✓ |  |  |  |  |  | ✓ |  |  |  |  |  |
| Control |  |  |  |  |  |  |  |  |  |  |  |  |  |  |  |  |  |
| 8 | ✓ |  |  |  | ✓ |  |  |  |  |  |  |  |  |  | ✓ |  |  |
| 9 |  |  |  |  | ✓ |  | ✓ |  | ✓ |  |  |  |  |  |  |  |  |
| 10 |  |  |  |  |  |  |  |  |  |  |  |  |  |  |  |  |  |
| 11 | ✓ |  |  |  | ✓ |  |  |  |  |  |  |  |  |  |  |  |  |
| 12 | ✓ |  | ✓ |  | ✓ | ✓ | ✓ | ✓ |  | ✓ | ✓ | ✓ |  |  |  |  | ✓ |
| 13 |  |  |  |  |  |  |  |  |  |  |  |  |  |  |  |  |  |
| 14 |  |  |  |  | ✓ | ✓ | ✓ | ✓ | ✓ |  | ✓ |  |  |  | ✓ |  |  |
| 15 |  |  |  |  | ✓ | ✓ | ✓ |  | ✓ | ✓ | ✓ |  |  |  |  |  |  |
| 19 |  | ✓ |  |  | ✓ | ✓ |  | ✓ | ✓ |  | ✓ |  |  |  |  |  |  |
| 20 |  |  |  |  |  |  |  |  |  |  |  |  |  |  |  |  |  |

| **Supplementary Table 3:** MINI International Neuropsychiatric Interview outcomes for disorders not previously listed for all participants. Blacked out boxes indicate an assessment did not take place | | | | | | | | | | | | | | | | | | | | | | | | |
| --- | --- | --- | --- | --- | --- | --- | --- | --- | --- | --- | --- | --- | --- | --- | --- | --- | --- | --- | --- | --- | --- | --- | --- | --- |
| Participant | OCD | | | | Compulsion | | | | Obsessions | | | | Alcohol Dependence | | | | Psychotic Disorders | | | | Pain Disorder | | | |
|  | Baseline | 3 months | 6 months | Baseline | | 3 months | 6 months | Baseline | | 3 months | 6 months | Past | Baseline | 3 months | 6 months | Past | | Baseline | 3 months | 6 months | | Baseline | 3 months | 6 months |
| iCBT |  |  |  |  | |  |  |  | |  |  |  |  |  |  |  | |  |  |  | |  |  |  |
| 1 | ✓ |  | ✓ |  | |  | ✓ | ✓ | |  |  | ✓ |  |  |  | ✓ | |  |  |  | | ✓ | ✓ | ✓ |
| 2 |  |  |  | ✓ | |  |  |  | |  |  |  |  |  |  |  | |  |  |  | | ✓ | ✓ | ✓ |
| 3 |  |  |  |  | |  |  |  | |  |  |  |  |  |  |  | |  |  |  | |  |  |  |
| 4 |  |  |  | ✓ | |  |  | ✓ | |  |  |  |  |  |  |  | |  |  |  | | ✓ |  |  |
| 5 |  |  |  |  | |  |  |  | |  |  |  |  |  |  |  | |  |  |  | |  |  | ✓ |
| 6 |  |  |  |  | |  |  |  | |  |  |  |  |  |  |  | |  |  |  | |  |  | ✓ |
| 7 |  |  |  | ✓ | |  |  |  | |  |  |  |  |  |  |  | |  |  |  | | ✓ |  |  |
| 16 | ✓ |  |  | ✓ | |  |  | ✓ | |  |  | ✓ |  |  |  | ✓ | | ✓ |  |  | | ✓ |  |  |
| 17 |  |  |  |  | |  |  |  | | ✓ |  |  |  |  |  |  | |  |  |  | |  | ✓ |  |
| 18 |  |  |  |  | |  |  |  | |  |  | ✓ |  |  |  |  | |  |  |  | | ✓ |  |  |
| Control |  |  |  |  | |  |  |  | |  |  |  |  |  |  |  | |  |  |  | |  |  |  |
| 8 |  |  |  | ✓ | | ✓ | ✓ | ✓ | | ✓ | ✓ |  |  |  |  |  | |  |  |  | |  |  |  |
| 9 | ✓ |  |  | ✓ | | ✓ | ✓ |  | |  |  |  |  |  |  |  | |  |  |  | | ✓ |  |  |
| 10 |  |  |  |  | | ✓ | ✓ |  | |  |  |  |  |  |  |  | |  |  |  | | ✓ |  |  |
| 11 |  |  |  |  | |  |  |  | |  |  |  |  |  |  |  | |  |  |  | |  |  |  |
| 12 |  |  |  |  | |  |  |  | |  |  | ✓ |  |  |  |  | |  |  |  | | ✓ | ✓ | ✓ |
| 13 |  |  |  |  | |  |  |  | |  |  |  |  |  |  |  | |  |  |  | |  |  |  |
| 14 |  |  |  |  | |  |  |  | |  |  |  |  |  |  |  | |  |  |  | |  |  | ✓ |
| 15 |  |  |  |  | |  |  |  | |  |  | ✓ |  | ✓ |  |  | |  |  |  | | ✓ | ✓ | ✓ |
| 19 |  |  |  | ✓ | |  | ✓ |  | |  |  |  |  |  |  |  | |  |  |  | |  |  |  |
| 20 |  |  |  |  | |  |  |  | |  |  |  |  |  |  |  | |  |  |  | |  |  |  |

| **Supplementary Table 4**: Scores for the assessments competed at baseline, 3-, and 6-months for those in the online iCBT intervention group and the control group. Scores are represented as mean(standard error). P-values are for group-time interaction. | | | | | | | |
| --- | --- | --- | --- | --- | --- | --- | --- |
| Assessment | Baseline | | 3 Months | | 6 Months | | P |
|  | iCBT | Control | iCBT | Control | iCBT | Control |  |
| *Psychiatric* |  |  |  |  |  |  |  |
| BDI | 20.0(6.08) | 15.8(2.78) | 14.6(5.20) | 12.5(3.01) | 14.1(5.01) | 17.5(4.97) | 0.067 |
| HAMA-D | 18.1(14.28) | 19.0(2.11) | 13.0(14.01) | 15.5(2.16) | 14.6(4.31) | 19.0(3.29) | 0.333 |
| GAD-7 | 8.71(2.35) | 8.25(1.86) | 5.71(2.42) | 7.00(1.83) | 8.00(3.17) | 7.62(2.56) | 0.675 |
| HAMA-A | 18.0(4.40) | 20.6(4.00) | 12.1(3.66) | 17.2(3.15) | 12.4(4.14) | 18.5(3.69) | 0.365 |
| *QoL* |  |  |  |  |  |  |  |
| SF-36 | 362(51.9) | 321(72.2) | 377(83.7) | 382(64.0) | 382(85.4) | 413(68.9) | 0.416 |
| *Motor* |  |  |  |  |  |  |  |
| BFMDRS | 13.7(3.19) | 10.6(3.12) | 13.6(3.50) | 9.97(3.02) | 13.0(3.77) | 9.91(2.98) | 0.880 |
| BDI, Beck’s Depression Inventory; BFMDRS, Burke-Fahn-Marsden Dystonia Rating Scale, GAD-7, Generalised Anxiety Disorder-7; HAM-A, Hamilton Scale for Anxiety; HAM-D, Hamilton Scale for Depression; iCBT, internet-based cognitive behavioural therapy; SF-36, Short Form-36 Health Survey | | | | | | | |
